# Regulation of Neurotrophin-3 and Interleukin-1**β** and Inhibition of Spinal Glial Activation Contribute to the Analgesic Effect of Electroacupuncture in Chronic Neuropathic Pain States of Rats

**DOI:** 10.1155/2015/642081

**Published:** 2015-06-16

**Authors:** Wenzhan Tu, Wansheng Wang, Haiyan Xi, Rong He, Liping Gao, Songhe Jiang

**Affiliations:** ^1^Department of Physical Medicine and Rehabilitation, The Second Affiliated Hospital & Yuying Children's Hospital of Wenzhou Medical University, Wenzhou, Zhejiang 325027, China; ^2^Department of Rehabilitation Medicine, The Affiliated Hospital of Binzhou Medical University, Binzhou, Shandong 256603, China; ^3^Department of Gynecology, The Affiliated Hospital of Binzhou Medical University, Binzhou, Shandong 256603, China

## Abstract

Growing evidence indicates that neurotrophin-3, interleukin-1*β*, and spinal glia are involved in neuropathic pain derived from dorsal root ganglia to spinal cord. Electroacupuncture is widely accepted to treat chronic pain, but the precise mechanism underlying the analgesic effect of EA has not been fully demonstrated. In this study, the mechanical withdrawal threshold and thermal withdrawal latency were recorded. We used immunofluorescence and western blots methods to investigate the effect of EA on the expression of NT-3 and IL-1*β* in DRG and spinal cord of CCI rats; we also examined the expression of spinal GFAP and OX-42 in spinal cord. In present study, the MWT and TWL of CCI group rats were lower than those in the Sham CCI group rats, but EA treatment increased the pain thresholds. Furtherly, we found that EA upregulates the expression of NT-3 in DRG and spinal cord of CCI rats, while EA downregulates the expression of IL-1*β*. Additionally, immunofluorescence exhibited that CCI-induced activation of microglia and astrocytes was inhibited significantly by EA treatment. These results demonstrated that the analgesic effect of EA may be achieved through promoting the neural protection of NT-3 as well as the inhibition of IL-1*β* production and spinal glial activity.

## 1. Introduction

Neuropathic pain, characterized by spontaneous pain, hyperalgesia, and allodynia, is often caused by peripheral nerve injury [1, 2]. Such pain is often persistent and poorly treated by existing therapies [3]. Now we know that neuroinflammation [4], purinergic signaling [5] and some other pain signaling molecules play key roles in the development of neuropathic pain. However, the neural protection and inhibition of spinal glia mechanism in treating neuropathic pain need be given more attention to.

Neurotrophin-3 (NT-3), a member of the neurotrophins, is a target-derived neurotrophic factor that regulates sensory neuronal survival and growth [6]. NT-3 is a potent negative modulator of the neuropathic pain state associated with chronic constriction injury (CCI) of the sciatic nerve [7], which can prevent the development and maintenance of thermal hyperalgesia [8]. Additionally, NT-3 delivered by exogenous administration has been reported to alleviate the mechanical hyperalgesia induced by intramuscular acid injection in transgenic mice [9]. Moreover, NT-3 can be produced by astrocytes [10] and microglia [11]. All these studies demonstrate the benefit of NT-3 on painful states.

Interleukin-1*β* (IL-1*β*), a polypeptide proinflammatory cytokine, plays an important role in modulating neuronal excitability in the peripheral nervous systems [12]. It is released under conditions associated with persistent pain including inflammatory pain and neuropathic pain [12]. Recent studies suggest that spinal IL-1*β* may be produced by glial cells (microglia and astrocytes) in different chronic pain states [13, 14]. Furthermore, there is growing recognition that spinal glia contributes to the development and maintenance of central sensitization in chronic pain [15].

Acupuncture has been used for more than 3000 years in traditional Chinese medicine [16]. Electroacupuncture (EA) is a procedure in which fine needles are inserted into an individual at discrete points, followed by electrical stimulation to relieve pain [17, 18]. In traditional Chinese medicine, Zusanli (ST-36) and Yanglingquan (GB34) are commonly used in acupuncture to treat neuropathic pain in the waist and lower extremities. ST36 is located 5 mm beneath the capitulum fibulae and lateral-posterior to the knee joint and GB34 is about 5 mm superior-lateral to ST36. ST36 and GB34 acupoints distribute near the common peroneal nerve and the superficial and deep peroneal nerves. Additionally, some studies [2, 19] have demonstrated that EA might alleviate neuropathic pain behavior of CCI rats and we have reported that EA could increase pain thresholds of rats with CCI [20]. When injury occurred, the nociceptive signals would form and come into the DRG neurons and then spinal dorsal horn following the corresponding nerves. Therefore, the analgesic effect of EA at ST36 and GB34 acupoints may be achieved by the regulation of NT-3, IL-1*β*, and spinal glia in DRG neurons or spinal dorsal horn.

The aim of the present study was to investigate whether the analgesic effect of EA was associated to following mechanism: (1) promote the neural protection of NT-3; (2) the anti-inflammatory effect by decreasing IL-1*β*; (3) inhibiting the activation of spinal glia.

## 2. Materials and Methods

### 2.1. Animals

The Institutional Animal Care and Use Committee of Wenzhou Medical University approved all experiments performed in accordance with the guidelines of the International Association for the Study of Pain. Male Sprague Dawley rats (200–250 g) were used for this study. The rats were randomly divided into 3 groups: Sham CCI group, CCI group, and CCI plus EA group. All animals were housed in plastic boxes at 22–24°C and provided free access to food and water under a 12/12 h reversed light-dark cycle.

### 2.2. Chronic Constriction Injury Model

The CCI model of neuropathic pain was chosen based on a previous description [21]. Briefly, after all rats were anesthetized with sodium pentobarbital (80 mg/kg, i.p.) and the right sciatic nerve was exposed at the mid-thigh level, proximal to the sciatic trifurcation, four ligature knots (4-0 chromic gut) were loosely tied with 1 mm intervals. In the Sham CCI group, the right sciatic nerve was exposed for 2-3 minutes but not ligated.

### 2.3. Mechanical Withdrawal Threshold (MWT)

In order to evaluate mechanical allodynia, the 2392 Electronic von Frey Anesthesiometer (IITC Life Science, USA) was applied to estimate the MWT. All rats were placed individually inside a wire mesh-bottom cages (20 cm × 14 cm × 16 cm) and given 20 min of adaptation. The probe was positioned below the plantar surface of the paw with von Frey filaments at a range of 0.1–70 g, with increasing force until the rat paw twitches. At the time of paw withdrawal, the maximum force was recorded. Each rat was tested alternately in 5 min intervals, and each rat was tested 6 times. Excluding the maximum and minimum forces, the average value was used as the MWT.

### 2.4. Thermal Withdrawal Latency (TWL)

In order to evaluate thermal hyperalgesia, the 37370 Plantar Test Apparatus (Ugo-Basile, Milan, Italy) was used to test the TWL. The rats were placed in a transparent acrylic chamber (17 cm × 11.5 cm × 14 cm) and given 20 min of adaptation. The radiant heat was set at 50°C and placed to the plantar surface of the hind paw. The withdrawal of the paw, indicating the sensation of pain in the rat, caused the infrared source stop and the reaction time was recorded. The hind paw was tested alternately at 10 min intervals and the cut-off time for heat stimulation was 40 s. Each rat was tested six times over the course of the experiment. Excluding the maximum and minimum times, the average value was expressed as the TWL.

### 2.5. Electroacupuncture (EA) Treatment

In the EA group, EA was started on day 7 after the CCI injury [22] and then given daily for the following 7 days; all EA was given between 9:00 and 11:00 a.m every day. The rats were maintained without anesthesia in an immobilization apparatus designed by our laboratory (patent application number: 201110021482.5, State Intellectual Property Office), a system convenient for acupuncture research and helpful to reduce stress for experimental rats. At ipsilateral ST-36 and GB-34, two needles were inserted to a depth of approximately 2-3 mm and connected to the output terminals of an EA apparatus (HANS-200E, Jisheng Medical Instruments). The frequency of stimulation was alternately applied as a square wave at 2/100 Hz, and the intensity of the stimulation was applied for 30 min at 2 mA.

### 2.6. Immunofluorescence

Half of all experimental animals were taken randomly for immunofluorescence study (*n* = 6 in each group). On day 14, the rats were deeply anesthetized using 5% chloral hydrate and perfused with 200 mL normal saline into the aorta, followed by 250 mL of 4% paraformaldehyde in 0.1 M phosphate buffered saline (PBS, pH 7.2–7.4). Subsequently, the ipsilateral L4-6 DRGs and whole L4-L5 lumbar spinal cords were removed, postfixed, and replaced with 30% sucrose. Transverse spinal sections (free-floating, 30 mm) and DRG sections (10 mm) were cut in a cryostat (Leica) and processed for immunofluorescence [23]. To ensure that immunohistochemical data were comparable between groups, free-floating sections were carefully processed by immunohistochemistry under the same conditions (such as the washing times, the incubating time, and the temperature). Followed by a PBS wash for 5 min, five times, all sections were sequentially blocked with 10% goat serum albumin, for one hour, in PBS + T (0.3% Triton-X 100) at room temperature and were incubated overnight at 4°C with different primary antibodies: rabbit polyclonal anti-NT-3 (1 : 200, Santa Cruz, USA), rabbit polyclonal anti-IL-1*β* (1 : 200, Santa Cruz, USA), mouse monoclonal anti-GFAP (astrocyte marker, 1 : 1000, Calbiochem, USA), and mouse monoclonal anti-OX-42 (microglia marker, 1 : 1000, ABD Serotec, USA). After the primary antibody incubation, the sections were then incubated for 1 h at room temperature with secondary antibodies (1 : 400, DyLight 488-labeled goat anti-rabbit IgG or DyLight 594 AffiniPure goat anti-mouse IgG, EarthOx, USA). Finally, sections were washed with PBS and the cover-slips were mounted onto slides using antifade mounting medium (Beyotime, China). The stained sections were examined with a fluorescence microscope (Olympus, Japan).

The quantitative analysis was performed on each animal from five randomly selected sections per animal. The immunofluorescence brightness and density of the staining were tested by Image Pro Plus software: the immunofluorescence density was used for examining the positive cell of NT-3 in DRGs; the immunofluorescence bright area was used for examining the expression of IL-1*β*, GFAP, and OX-42 in spinal cord.

### 2.7. Western Blots

The remainder of experimental animals were used for western blots (*n* = 6 in each group). On day 14 after the CCI operation, the rats were deeply anesthetized and the L4-L5 spinal cord segments were isolated immediately and flushed with ice-cold PBS. The segments were lysed and microfuged at 12,000 rpm for 5 min at 4°C, and subsequently the supernatant was collected. Protein samples (30 *μ*g) were loaded on a 10% Tris–HCl SDS-PAGE gel (Bio-Rad, Hercules, CA) for 30 min at 70 V and 55 min at 120 V. After electrophoresis, the proteins were electrotransferred to a polyvinylidene fluoride (PVDF) membrane for 50 min at 300 mA. The membranes were blocked with Tris-buffered saline (TBS), containing 0.1% Tween-20, 5% skim milk, and 0.2% BSA for 2 h at room temperature and incubated over night at 4°C with primary antibodies: anti-NT-3 (1 : 300, Santa Cruz, USA) and anti-IL-1*β* (1 : 250, Santa Cruz, USA). The membranes were washed four times with TBST and incubated (1.5 h, room temperature) with horseradish peroxidase-conjugated secondary antibody (goat anti-rabbit IgG 1 : 5000, Chemicon, USA) in blocking buffer. After being washed, the labeled proteins were visualized using the enhanced chemiluminescence (ECL) kit (Beyotime, China). The immune complex was collected on Kodak light film and the quantity of band intensity was detected by a DNR Micro Chemi Chemiluminescence gel imaging system. The band densities were normalized to each glyceraldehyde-3-phosphate dehydrogenase (GAPDH).

### 2.8. Statistical Analysis

All results were expressed as the means ± standard deviation (SD). The statistical differences were analyzed using one-way ANOVA with Tukey or Dunnett's post hoc tests for multiple comparisons. A *P* value < 0.05 was considered statistically significant.

## 3. Results

### 3.1. Effect of EA on Mechanical and Thermal Hyperalgesia of CCI Rats

**Figure 1:**
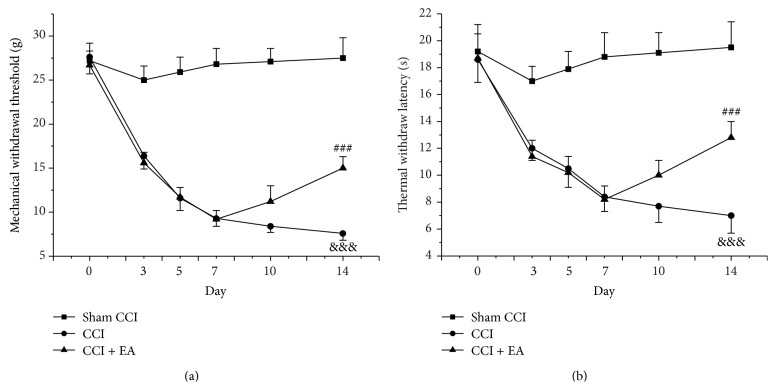
Analgesic effects of EA treatment on mechanical withdrawal threshold (MWT) and thermal withdrawal latency (TWL) induced by chronic constrictive injury. On day 14, the MWT (a) and TWL (b) in each group were recorded and compared with each other. ^&&&^ *P* < 0.001, versus the Sham group; ^###^ *P* < 0.001, versus the CCI group.

Before and on days 3, 5, 7, 10, and 14 after the CCI operation, the MWT and TWL were measured. On day 14, the MWT and TWL in the CCI group were significant lower than those in the Sham CCI group (*F*_(2,21)_ = 458.4, *P* < 0.001; *F*_(2,21)_ = 144.6, *P* < 0.001). However, the MWT and TWL in the EA group were higher than those in CCI group (*P* < 0.001, *P* < 0.001), implying EA treatment could increase the mechanical and thermal threshold in the rats suffering from neuropathic pain after CCI operation (Figures 1(a) and 1(b)).

### 3.2. Effect of EA on the Immunoreactive Changes of NT-3 in DRGs

**Figure 2:**
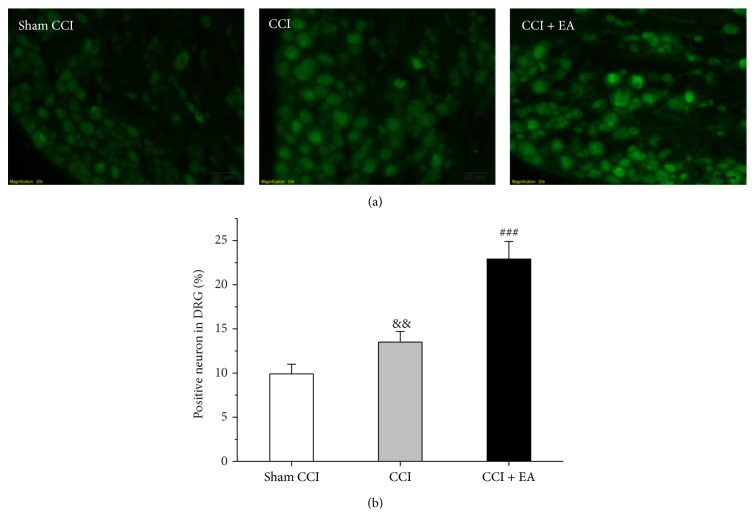
Effect of EA on CCI-induced increase of the immunoreactive changes of NT-3 in DRG. (a) NT-3 immunopositive neurons in ipsilateral DRG of each group. (b) Quantification of positive neurons showing that EA treatment promoted the expression of NT-3. ^&&^ *P* < 0.01, versus the Sham group; ^###^ *P* < 0.001, versus the CCI group.

The expression of NT-3 in DRG was observed through immunofluorescence. The immunofluorescence density >12 was used for examining positive cells. In the CCI group, the number of positive neurons was more than that in the Sham CCI (*F*_(2,15)_ = 61.1, *P* < 0.001). However, after EA treatment, the expression of NT-3 increased further (*P* < 0.001; versus the CCI group) (Figures 2(a) and 2(b)).

### 3.3. Effect of EA on the Quantitative Changes of NT-3 Protein in Spinal Cord

**Figure 3:**
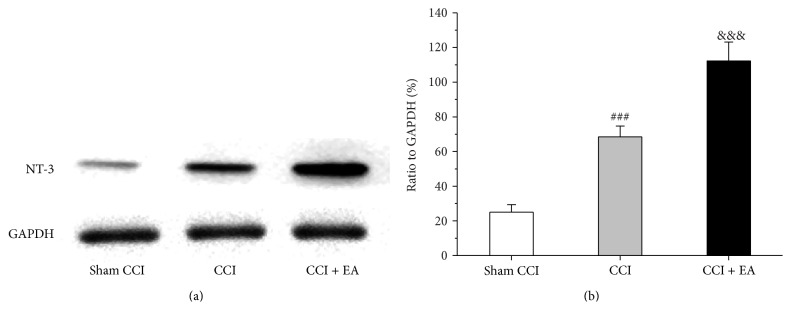
Effect of EA on CCI-induced increase of the quantitative changes of NT-3 protein in spinal cord. (a) Changes in the relative content of NT-3 protein in spinal cord of every group. (b) Quantification of bands showing that EA treatment promoted the expression of NT-3. ^###^ *P* < 0.001, versus the Sham group; ^&&&^ *P* < 0.001, versus the CCI group.

The NT-3 expression at the protein level in spinal cord was analyzed using western blotting. The relative optical density (ROD) value for the NT-3 protein expression in the CCI group was significantly higher than that in Sham CCI group (*F*_(2,15)_ = 96.2, *P* < 0.001), and EA treatment increased that ROD in the EA group. Similar to the result of immunohistochemistry, the level of NT-3 protein significantly increased after EA treatment (*P* < 0.001) (Figures 3(a) and 3(b)).

### 3.4. Effect of EA on the Immunoreactive and Quantitative Changes of IL-1*β* in Spinal Cord

**Figure 4:**
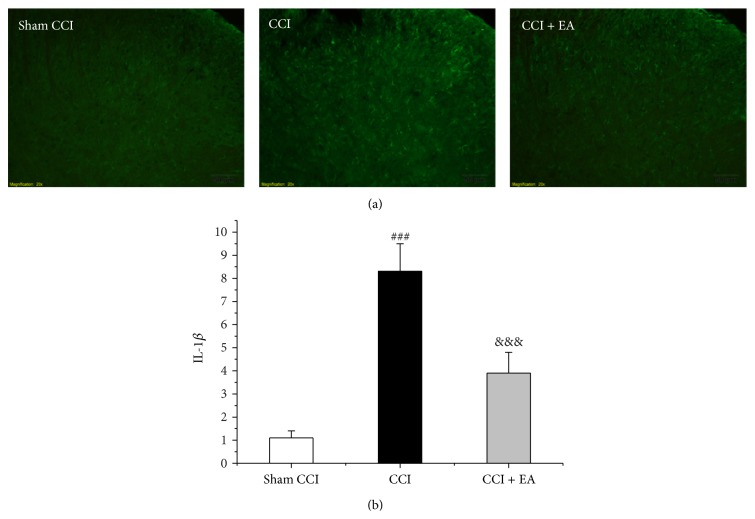
Effect of EA on CCI-induced increase of the immunoreactive of IL-1*β* in spinal cord. (a) Results of IL-1*β* immunofluorescence in ipsilateral spinal dorsal horn. (b) Changes of the positive area showing that EA treatment suppressed the expression of IL-1*β*. ^###^ *P* < 0.01, versus the Sham group; ^&&&^ *P* = 0.001, versus the CCI group.

**Figure 5:**
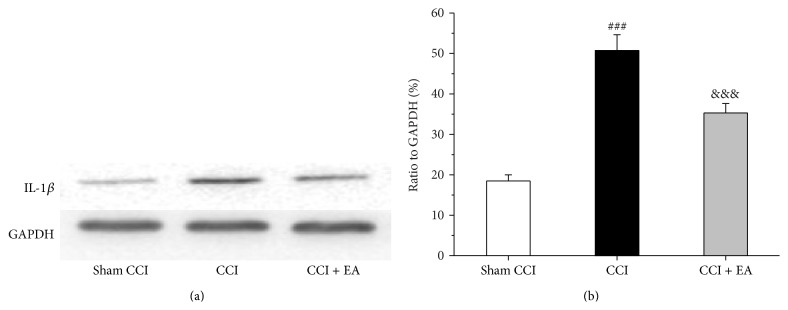
Effect of EA on the increase of IL-1*β* protein induced by CCI in spinal cord. (a) Results of western blot from spinal cord in each group. (b) Changes in the relative content of IL-1*β* protein from picture (a). ^###^ *P* < 0.01, compared with the Sham group; ^&&&^ *P* < 0.001, compared with the CCI group.

The expression of IL-1*β* in spinal cord was observed through immunofluorescence and western blotting. CCI injury increased the expression of IL-1*β* in spinal cord (*F*_(2,15)_ = 50.2, *P* < 0.001; *F*_(2,15)_ = 144.6, *P* < 0.001), compared with the Sham CCI group (Figures 4 and 5). However, in the CCI + EA group, the immunoreactivity and quantitativeness of protein of IL-1*β* were lower than those in the CCI group (*P* < 0.001, *P* < 0.001), implying EA could inhibit the expression of IL-1*β* in spinal cord of CCI rats.

### 3.5. Inhibitory Effect of EA on Spinal Glial Activation

**Figure 6:**
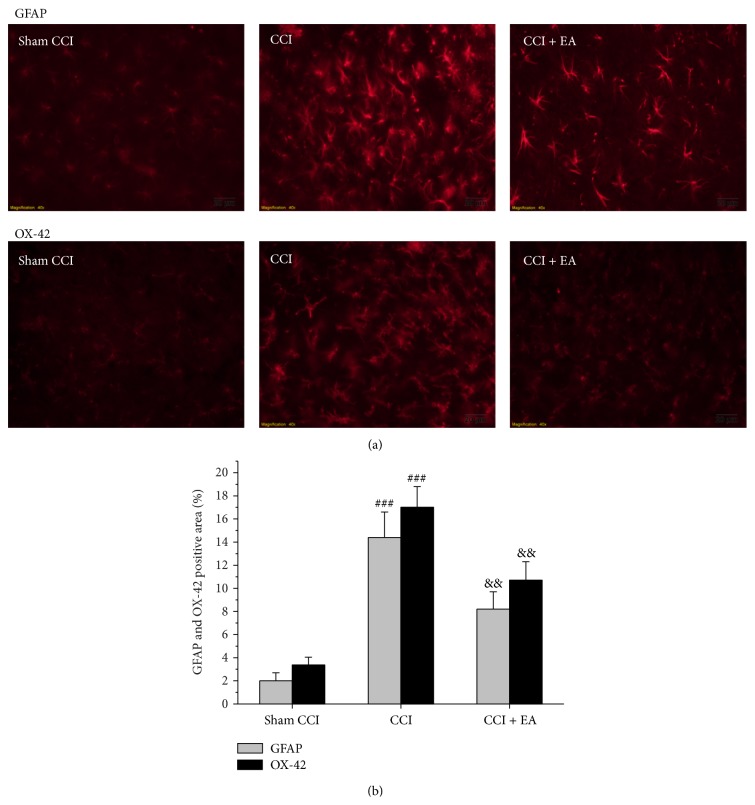
Effect of EA on CCI-induced increase of the immunoreactivity of GFAP and OX-42 in the spinal dorsal horn. (a) Results of GFAP and OX-42 immunofluorescence in ipsilateral spinal dorsal horn. (b) Changes of the positive area from picture (a) showing that EA treatment suppressed the expression of GFAP and OX-42 in spinal cord. ^###^ *P* < 0.001 and ^###^ *P* < 0.001, versus the Sham group; ^&&^ *P* = 0.002 and ^&&^ *P* = 0.003, versus the CCI group.

By means of immunohistochemistry, we used GFAP and OX-42 to label astrocyte and microglia in the spinal cord, respectively. Expression of GFAP and OX-42 was obviously upregulated in the spinal dorsal horn on day 14 after CCI injury (*F*_(2,15)_ = 44.4, *P* < 0.001 and *F*_(2,15)_ = 67.48, *P* < 0.001, versus Sham CCI group). The expression of GFAP and OX-42 in CCI + EA group was lower than that in CCI group (*P* = 0.002 and *P* = 0.003). This result indicated that EA could inhibit the activation of astrocyte and microglia induced by CCI (Figures 6(a) and 6(b)).

## 4. Discussion

NT-3 is a target-derived neurotrophic factor that regulates sensory neuronal survival and growth [6]. It has been reported NT-3 could prevent the development and maintenance of thermal hyperalgesia with CCI of the sciatic nerve [8]. So, NT-3 may release the neural plasticity caused by CCI and decrease the neurocells sensibility to stimulation. Here we have shown that the pain hypersensitivity of CCI rats was released and NT-3 protein was upregulated in DRG and spinal cord after EA treatment. These findings suggest that NT-3 could promote the analgesia effect of EA in neuropathic pain.

A growing body of evidence implicates that spinal glia was involved in the modulation of chronic pain [24] and EA analgesia [15, 25]. Painful syndromes are associated with different glial activation states: glial reaction (i.e., upregulation of glial markers such as glial fibrillary acidic protein (GFAP) and OX-42 and/or morphological changes, including hypertrophy and proliferation) [26]. In parallel with these reports, the present study showed that CCI promotes the glial reaction, whereas EA inhibits the reaction. Furthermore, it has been demonstrated that knockdown of NT-3 markedly increased the expression of GFAP, OX-42 in the spinal dorsal horn during inflammatory pain [15]. This finding suggests that the antihyperalgesic role of NT-3 in neuropathic pain may be mediated through the inhibition of glial activity.

Spontaneous and evoked pain after nerve injury are thought to derive from hyperexcitability of primary and/or secondary afferent neurons generated by neurotrophins and proinflammatory cytokines released from activated inflammatory cells including microglia [27, 28] that become activated in the dorsal horn, astrocytes [29], and macrophages that invade the lesion site [30]. IL-1*β* is a cytokine released from spinal glial cells in response to pathophysiological changes that occur during different disease states, such as neuropathic pain and inflammatory [12, 31]. Initial reports suggested that IL-1*β* is an extremely potent hyperalgesic agent when injected systemically, intraperitoneally, or intraplantarly in rats [32]. In addition, the hypothalamic and ventral midbrains mRNA levels of IL-1*β* raised by inflammation could be reversed to normal levels by acupuncture stimulation [33]. Now that glia plays an important role in nociceptive transmission in neuropathic pain [34] and glial activity would be inhibited by NT-3; the synthesis and secretion of proinflammatory cytokines from glial cells would be decreased too. Corresponding with this conclusion, we also found the upregulation of IL-1*β* in spinal cord in CCI rats was suppressed after EA treatment.

In recent years, we have changed it as follow: more and more attention given to the possible roles of neurotrophins and cytokines in the therapeutic effects of acupuncture. The cross talk of neurotrophins and cytokines from peripheral nervous system (PNS) to central nervous system (CNS) is involved in the pathophysiology of many human diseases and may contribute to the effects of acupuncture [35]. Therefore, the relationship between NT-3 and IL-1*β* may be an important contributor to chronic pain mechanism. It has been reported that IL-1*β* could act on sensory neurons to increase their susceptibility for injury [36, 37], while NT-3 might release the neural plasticity caused by nerve injury and decrease the neurocells sensibility to stimulation. Interestingly, it has been demonstrated that IL-1*β* could upregulate the expression of NT-3 [38]. Based on these findings, we thought that IL-1*β* might increase the expression of NT-3 at the start of neuropathic pain, while NT-3 might depress the expression of IL-1*β* at the following stage. Indeed, EA treatment was given on day 7 after CCI injury in this study. So, EA may have an anti-inflammatory effect through upregulating the expression of NT-3. This conclusion is in accordance with the study that demonstrated that NT-3 might serve as an anti-inflammatory factor to suppress neuropathic pain [39].

The relationship between NT-3, IL-1*β*, and spinal glial cell is very important to explain the analgesic effect of EA in neuropathic pain states of rats now. Recently, it was reported that antisense oligodeoxynucleotides specifically against NT-3 intrathecally administered could suppress expression of spinal GFAP, OX-42, and proinflammatory cytokines stimulated with arthritis [15]. In addition, the inhibition of proinflammatory mediators by NT-3 pretreatment in primary microglia with LPS stimulation was corroborated [40]. Based on these published reports and the result of our study, we thought NT-3 may be involved in the analgesic effect of EA on neuropathic pain states of rats mediated through the inhibition IL-1*β* production and spinal glial activity.

The perception of pain requires the activation of multiple neurons across the pain system and the interactions between the thalamus, cortex, and limbic system [41]. When injury occurs, the peripheric receptor is activated and nociceptive signals are carried from the periphery to the dorsal horn of the spinal cord mostly by two populations of small diameter primary afferents, the peptidergic and the nonpeptidergic [42]. The peptidergic population expresses neuropeptides, such as substance P and calcitonin gene-related peptide, while the nonpeptidergic fibers are devoid of neuropeptides, express the purinergic receptor P2X_3_, and bind the isolectin B4 (IB4) [42]. Considerable studies have demonstrated that chronic constriction injury of the sciatic nerve induces persistent pain behaviors in rats [43, 44] and purinergic signaling has been proved to be implicated in neuropathic pain [43, 45, 46]. Additionally, our previous study [20] has proved that EA might increase the pain thresholds through downregulating the expression of P2X_3_ receptor. It is a truth that some purinergic receptors (e.g., P2X_4_ and P2X_7_) are coexpressed with spinal glial. So, following the inhibition of glial activity, purinergic signaling would be inhibited too. A previous study has also shown that peripheral P2X receptors are involved in mediating the peripheral excitation of C- and A*δ*-fiber [47]. Importantly, the ability of NT-3 to prevent and reverse thermal hyperalgesia, believed signaled by C-fibers, is a novel finding with respect to modulation of neuropathic pain [48]. In addition, the involvement of C-type afferents in EA analgesia has been proved [17]. Therefore, we speculated that NT-3 may depress purinergic signals in the analgesic effect of EA, which needs further study.

Based on previous [20] and the present study, we thought that the neuroprotective effect of NT-3 plays a key role in the analgesic effect of EA. The incremental NT-3 inhibited the activation states of spinal glia and downregulated the expression of IL-1*β*. All these changes promote the stability of neurocells and decrease the generation of pain signals which is transmitted through C-fiber by purinergic signaling.

In conclusion, the present study provided new evidences that EA may exert analgesic effect by inhibiting the sprouting of nociceptive signals. Further, this effect may be achieved through the neural protection of NT-3, which decreases the expression of IL-1*β* and the activation of spinal glia. These results of this study provide a new and promising understanding about the mechanism underlyingthe analgesic effect of EA.
